# Supplementary material for: Facile Synthesis of GNPs@NixSy@MoS2 Composites with Hierarchical Structures for Microwave Absorption
Source: Nanomaterials (Basel). 2019 Oct 2;9(10):1403. doi: 10.3390/nano9101403 (PMC6835756; doi:10.3390/nano9101403)
Supplement: Supplementary file 1 [file nanomaterials-09-01403-s001.pdf]

Supporting Information

Facile Synthesis of GNPs@Ni_xS_y@MoS_2 Composites with Hierarchical Structures for Microwave Absorption

Wenfeng Zhu, Li Zhang, Weidong Zhang*, Fan Zhang, Zhao Li, Qing Zhu, and Shuhua Qi*

Department of Applied Chemistry, School of Natural and Applied Sciences, Northwestern Polytechnical University, Xi'an 710072, China; zwenfeng89@mail.nwpu.edu.cn (W.Z.); zhangli_nwpu@outlook.com (L.Z.); jofun@mail.nwpu.edu.cn (F.Z.); lizhao1314@mail.nwpu.edu.cn (Z.L.); zhuqing@mail.nwpu.edu.cn (Q.Z.)

* Correspondence: weidzhang1208@126.com (W.Z.); qishuhuanwpu@163.com (S.Q.); Tel.: 86-13186052872 (W.Z.); 86-13659243868 (S.Q.)

Received: 18 September 2019; Accepted: 26 September 2019; Published: date

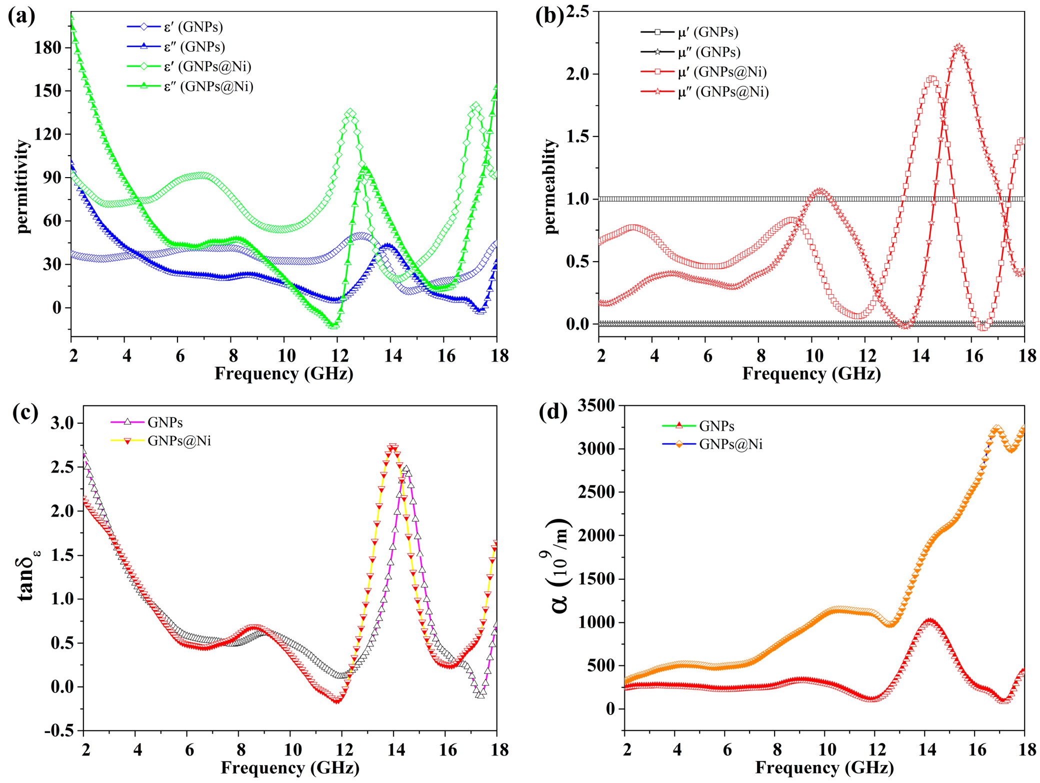

Figure S1. Electromagnetic parameters (a, b), tangent of dielectric loss (c) and α (d) of of GNPs and GNPs@Ni composite.

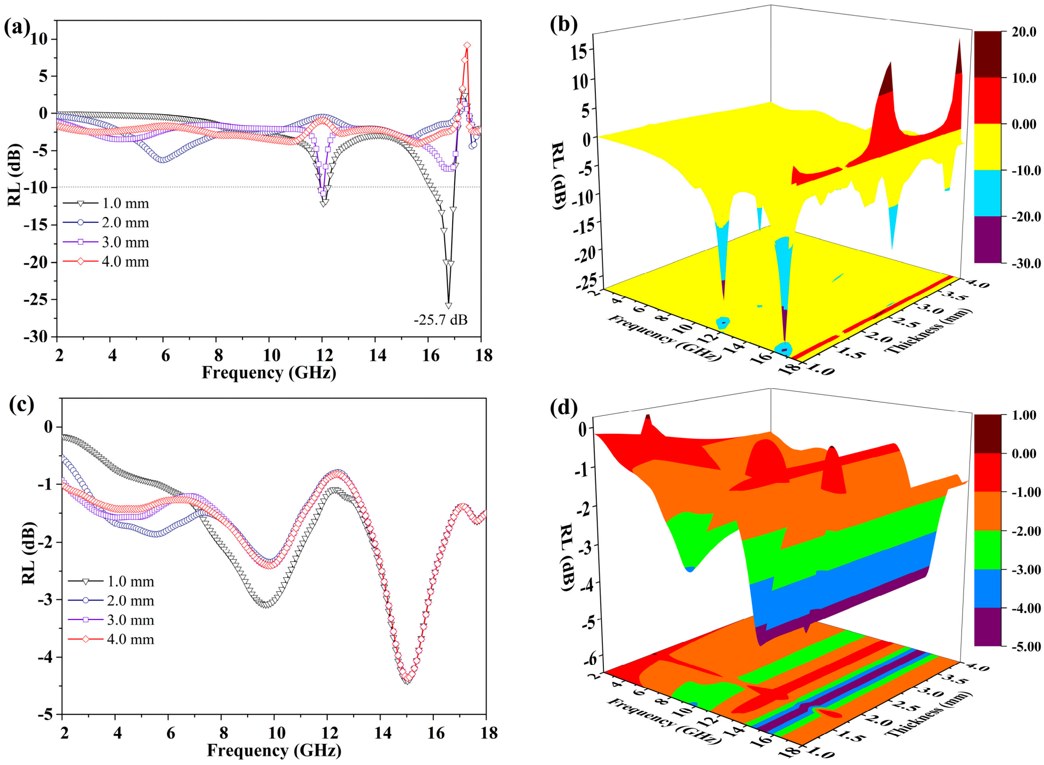

Figure S2. RL and corresponding 3D plots of GNPs (a, b) and GNPs@Ni composite (c, d).

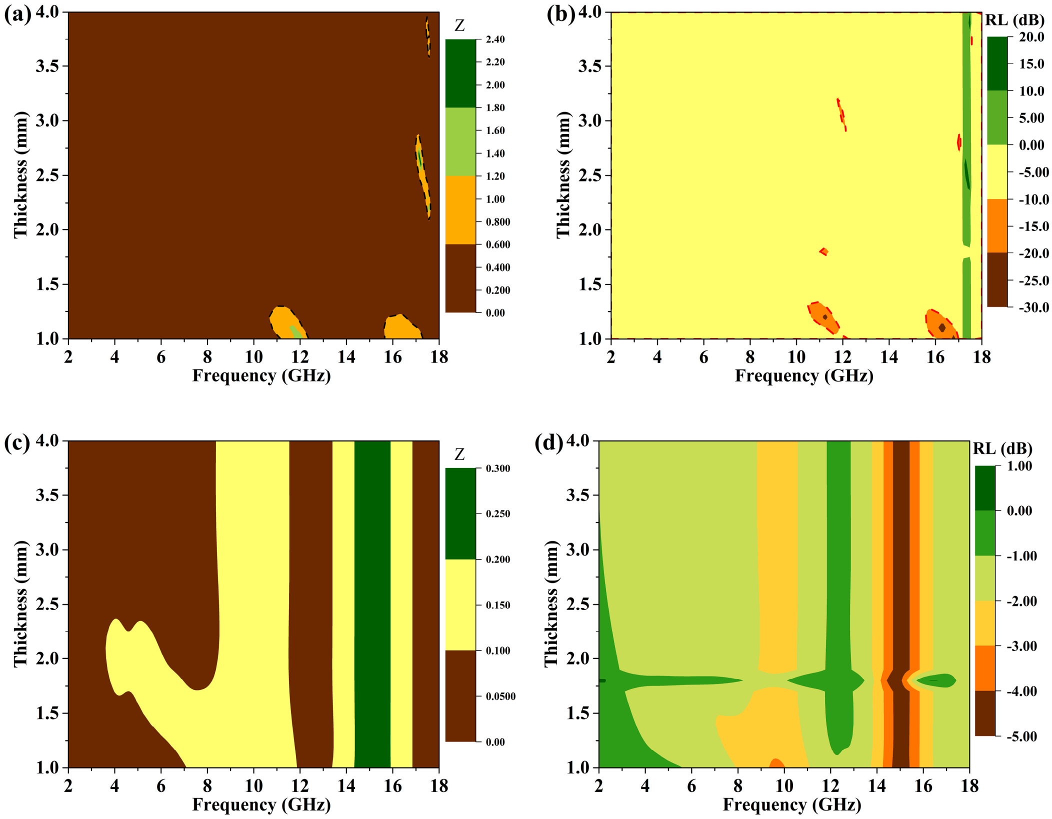

Figure S3. Z and RL contour maps of GNPs (a, b) and GNPs@Ni composite (c, d).

Table S1. The composition of as-prepared composites.

Samples	GNPs (%)	Ni (%)	Ni_xS_y (%)	MoS_2 (%)
GNPs@Ni	62	38	0	0
GNPs@Ni_xS_y	83.3	0	16.7	0
GNPs@Ni_xS_y@MoS_2-1	37.8	0	5.47	56.73
GNPs@Ni_xS_y@MoS_2-2	37.1	0	5.38	57.52
GNPs@Ni_xS_y@MoS_2-3	37.2	0	5.39	57.41

Table S1. Conductivity of as-prepared GNPs, GNPs@Ni and GNPs@Ni_xS_y@MoS_2 nanocomposites.

Samples	GNPs	GNPs@Ni	GNPs@Ni_xS_y@MoS_2-1	GNPs@Ni_xS_y@MoS_2-2	GNPs@Ni_xS_y@MoS_2-3
Conductivity (S/cm)	301.2	1612.2	3.3344	1.7177	0.5258
